# Long-Term Outcomes of Heart Failure Patients With Preserved, Mildly Reduced, and Reduced Ejection Fraction

**DOI:** 10.1016/j.jacasi.2022.11.013

**Published:** 2023-03-07

**Authors:** Takuya Nagata, Tomomi Ide, Takeshi Tohyama, Hidetaka Kaku, Nobuyuki Enzan, Shouji Matsushima, Masataka Ikeda, Koji Todaka, Hiroyuki Tsutsui

The long-term prognosis of heart failure (HF) in Japan, a country with a long-life expectancy and an aging population, has not been fully elucidated in recent years. Most epidemiological studies about HF have focused on HF with reduced ejection fraction (HFrEF) (left ventricular ejection fraction [LVEF] <40%) or HF with preserved ejection fraction (HFpEF) (LVEF ≥50%), and a few have focused on patients with HF with mildly reduced ejection fraction (HFmrEF) (LVEF 40%-49%).

The JROADHF (Japanese Registry Of Acute Decompensated Heart Failure) is a nationwide, multicenter, retrospective observational study of hospitalized patients with HF.^1^ Subjects for the JROADHF study were selected by cluster random sampling from the JROAD-DPC (Japanese Registry Of All Cardiac and Vascular Diseases-Diagnosis Procedure Combination) study, a comprehensive registry of acute care facilities in Japan.^2^ The total number of patients in JROADHF was 13,238. After excluding 1,665 patients with missing LVEF data assessed by echocardiography at admission, 748 patients who had died in the hospital, and 930 patients with missing follow-up data, 9,895 patients were included. Subjects were followed retrospectively from the day of discharge in 2013 to December 2017. The median follow-up was 4.3 years (IQR: 3.8-4.7 years), and the follow-up rate was 91.4%. Age- and sex-adjusted mortality rates across LVEF categories were calculated using the direct method (calculating the standardized rate of the study population as a weighted average of the stratification rates using weights from the reference population)^3^ and Cox proportional hazards model (estimating survival curves corresponding to mean age and frequency of female),^4^ respectively. The JROADHF protocol was approved by the Clinical Research Ethics Review Committee of the Kyushu University Medical District Department and all 128 participating institutions.

**Figure 1:**
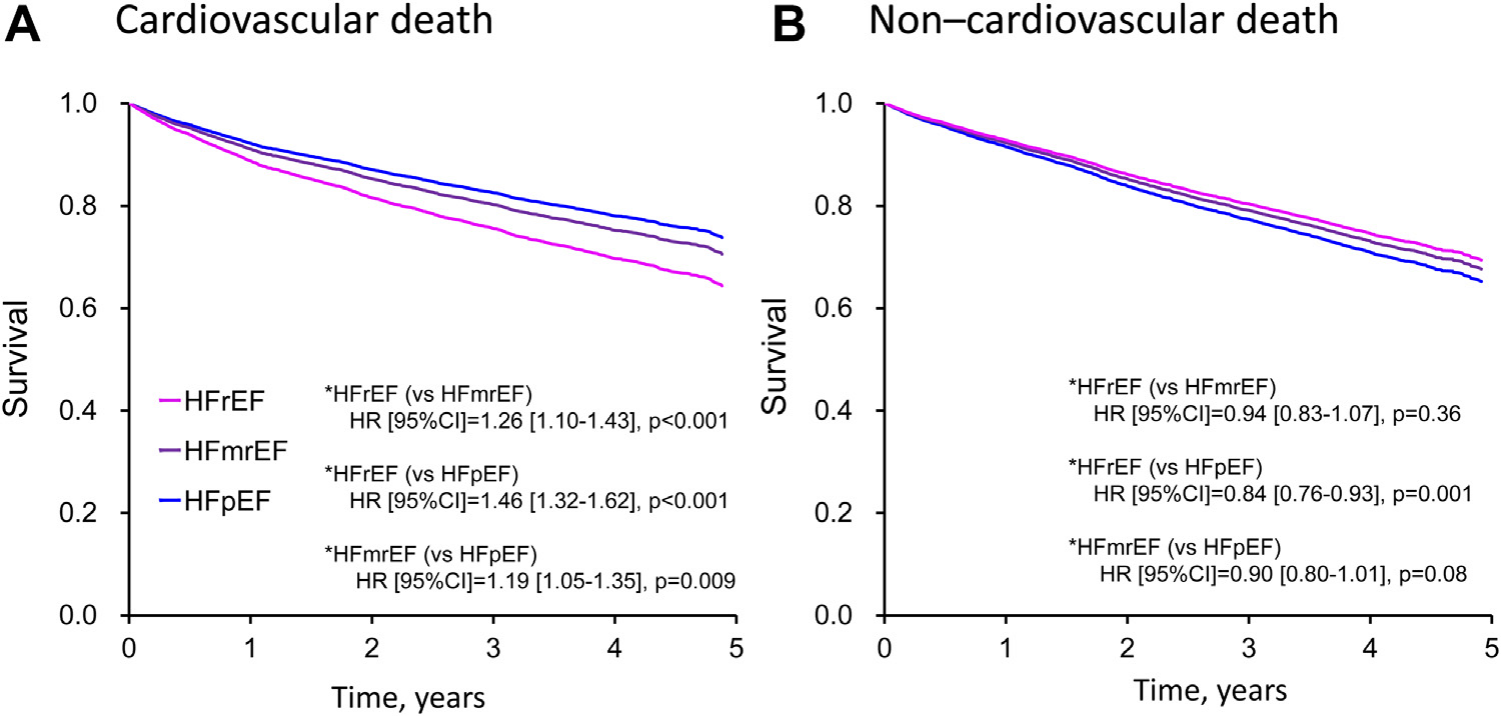
Survival Rates of Cardiovascular and Noncardiovascular Death by Ejection Fraction Age- and sex-adjusted survival rates of **(A)** cardiovascular death and **(B)** noncardiovascular death according to left ventricular ejection fraction categories. HFmrEF = heart failure with mildly reduced ejection fraction; HFpEF = heart failure with preserved ejection fraction; HFrEF = heart failure with reduced ejection fraction.

The age- and sex-adjusted all-cause mortality rates of HFrEF, HFmrEF, and HFpEF were 16.8, 16.1, and 15.9 per 100 person-years, respectively, and the risks were significantly higher in HFrEF than in HFpEF (HFrEF vs HFpEF: HR: 1.10; 95% CI: 1.03-1.19; *P* = 0.007). When divided into cardiovascular and noncardiovascular deaths, the age- and sex-adjusted mortality rates for HFrEF, HFmrEF, and HFpEF were 9.2, 7.8, and 6.6 per 100 person-years for cardiovascular death and 7.6, 8.3, and 9.3 per 100 person-years for noncardiovascular death, respectively. The risk of cardiovascular death was significantly higher in HFrEF than in HFpEF (HR: 1.46; 95% CI: 1.32-1.62; *P* < 0.001). Conversely, the risk of noncardiovascular death was significantly lower in HFrEF than in HFpEF (HR: 0.84; 95% CI: 0.76-0.93; *P* = 0.001) (Figure 1). Of the 1,472 patients who developed noncardiovascular death, 443 (30.1%) died of pneumonia, and 372 (25.3%) died of cancer.

The GWTG (Get With The Guidelines) study,^5^ the Swedish Heart Failure Study,^6^ and the CHART-2 (Congestive Heart Failure Cardiopoietic Regenerative Therapy) study^7^ reported a slightly worse prognosis in HFrEF and a similar prognosis in HFmrEF and HFpEF. These findings agree with the present study. In this study, approximately half of the patients with HF died of noncardiovascular causes, a higher percentage than in previous studies in Japan.^8^ This might be because our subjects are older, and this cohort covered community hospitals as well as advanced treatment hospitals.
